# Admission serum myoglobin and the development of acute kidney injury after major trauma

**DOI:** 10.1186/s13613-021-00924-3

**Published:** 2021-09-24

**Authors:** Virginie Tarazona, Samy Figueiredo, Sophie Hamada, Jonas Pochard, Ryan W. Haines, John R. Prowle, Jacques Duranteau, Bernard Vigué, Anatole Harrois

**Affiliations:** 1 Department of Anesthesiology and Critical Care, Bicêtre Hospital, Assistance Publique-Hôpitaux de Paris (AP-HP), DMU 12 “Anesthésie-Réanimation-Douleur”, Université Paris Saclay, 78 rue du Général Leclerc, 94275 Le Kremlin Bicêtre, France; 2 Adult Critical Care Unit, The Royal London Hospital, Barts Health NHS Trust, Whitechapel Road, London, E1 1BB UK; 3 William Harvey Research Institute, Queen Mary University of London, London, UK

**Keywords:** Trauma, Acute kidney injury, Rhabdomyolysis, Myoglobin, Creatine-kinase

## Abstract

**Background:**

Myoglobin and creatine kinase (CK) are both established markers of muscle injury but their hospital admission values have never been compared to predict post-traumatic acute kidney injury (AKI).

**Methods:**

An observational registry study of consecutive trauma patients admitted to a major regional trauma centre. The primary outcome was stage 1 or more AKI in the first 7 days after trauma. We assessed the association of hospital admission myoglobin or CK with development of AKI both alone and when added to two existing risk prediction models for post traumatic AKI.

**Results:**

Of the 857 trauma patients (median age 36 [25–52], 96% blunt trauma, median ISS of 20 [12–47]) included, 102 (12%) developed AKI. Admission myoglobin performed better than CK to predict AKI any stage with an AUC–ROC of 0.74 (95% CI 0.68–0.79) and 0.63 (95% CI 0.57–0.69), respectively (*p* < 0.001). Admission myoglobin also performed better than CK to predict AKI stage 2 or 3 [AUC–ROC of 0.79 (95% CI 0.74–0.84) and 0.74 (95% CI 0.69–0.79), respectively (*p* < 0.001)] with a best cutoff value of 1217 µg/L (sensitivity 74%, specificity 77%). Admission myoglobin added predictive value to two established models of AKI prediction and showed significant ability to reclassify subjects regarding AKI status, while admission CK did not. Decision curve analysis also revealed that myoglobin added net benefit to established predictive models. Admission myoglobin was better than CK at predicting development of significant rhabdomyolysis.

**Conclusions:**

Admission myoglobin better predicts the development of AKI and severe rhabdomyolysis after major trauma. Admission myoglobin should be added in established predictive models of post-traumatic AKI to early identify high-risk patients.

**Supplementary Information:**

The online version contains supplementary material available at 10.1186/s13613-021-00924-3.

## Background

Rhabdomyolysis results from muscle injury that leads to the release of intramuscular content into the systemic circulation. Severe trauma patients are particularly susceptible to rhabdomyolysis, owing to a variety of muscle insults, such as blunt trauma, poor muscle perfusion, occasional limb ischemia or muscle compartment syndrome. Moreover, prolonged repair surgeries are commonly required after severe trauma and further worsen rhabdomyolysis by causing a “second traumatic hit” to muscles [1]. Of note, several studies have reported rhabdomyolysis to be a risk factor for post-traumatic acute kidney injury (AKI) [2–4] that is independently associated with increased mortality [4]. Thus, the early identification of trauma-induced rhabdomyolysis would enable the initiation of prophylactic interventions to limit AKI [5].

Creatine kinase (CK) and myoglobin are both established biomarkers to diagnose and monitor rhabdomyolysis severity [6]. Many studies have reported an association between maximum CK or myoglobin measured during hospital stay and AKI [2, 3, 7–10]. However, CK and myoglobin peaks are late markers of rhabdomyolysis severity, which makes them inappropriate to predict AKI early. To overcome this, some studies focused on admission CK [11–13] and myoglobin [14] to predict post-traumatic AKI. However, these studies were not limited to trauma patients and did not compare the ability of both biomarkers measured on admission to predict post-traumatic AKI. Based on a small-sample, prospective cohort study (communication, Congress of the French Society of Anaesthesiology and Intensive Care 2017) from our department, we hypothesized that admission myoglobin would have a greater association with occurrence of AKI than admission CK after severe trauma.

To test this hypothesis, we conducted a study aimed to answer the following questions: (1) which of admission CK and myoglobin performs best to predict AKI after trauma, (2) which of these two biomarkers adds predictive value in established models of post-traumatic AKI prediction and (3) which of these two variables performs best to predict severe rhabdomyolysis.

## Methods

This retrospective study was conducted in the department of anesthesia and surgical intensive care of Bicêtre University hospital (Paris, France) between January 2015 and June 2017. Data were prospectively collected in the “traumabase” registry, a French registry that includes 25 centres across the national territory. In this study, we only used data from Bicêtre centre. We obtained approval for the use of data collected in the registry, including waived informed consent from the Institutional Review Board (Comité pour la Protection des Personnes, Paris VI-Pitié-Salpêtrière, France). The database was approved by the Advisory Committee for Information Processing in Health Research (Comité Consultatif sur le Traitement de l’Information en matière de Recherche dans le Domaine de la Santé, CCTIRS 11.305bis), and the French National Commission on Computing and Liberty (Commission Nationale Informatique et Liberté, CNIL 911461). Retrospective collection of additional data was approved by the Institutional Review Board (Comité d’Ethique de la Recherche en Anesthésie-Réanimation, CERAR, IRB00010254‐2019-130).

### Study population

We included all consecutive trauma patients above 18 years, directly admitted from the scene to the study centre, having a measure of serum myoglobin and CK performed on admission and having serum creatinine measured to assess renal function. The Paris emergency medical system and trauma management have been described elsewhere [15]. Patients suspected of severe trauma (according to the presence of at least one Vittel triage criterion as assessed by the physician on scene, see Additional file 1) were directly admitted to the trauma resuscitation room of the study centre.

### Data collection

Hemodynamic, respiratory and neurologic variables in the prehospital setting and on arrival at the hospital were collected. On admission, blood was immediately sampled for blood typing and analysis that included blood gas, lactate, creatine kinase (CK), myoglobin and coagulation tests. Biological samples were repeated thereafter to monitor rhabdomyolysis at the discretion of clinicians. In vitro quantitative determination of myoglobin in serum was performed with a two-step sandwich immunoassay (Elecsys Myoglobin assay, Cobes analyser, Roche, Meylan, France). CK measurement was performed by assessing creatine-kinase activity in the presence of d-glucose (Ultra violet test, Roche Diagnostics, Switzerland). Maximum myoglobin and CK were defined as the maximum values measured over the first 7 days of admission. Clinical severity was assessed using the following severity scores: Simplified Acute Physiology Score II (SAPS II) and Sequential Organ Failure Assessment (SOFA). The severity of traumatic injuries was assessed using the Abbreviated Injury Scale (AIS) 1998 version and the Injury Severity Score (ISS). The trauma-related injury severity score (TRISS) was also determined [16, 17]. ICU length of stay and mortality were reported.

All the patients admitted to our centre had their data recorded by dedicated research technicians. Algorithms for consistency and coherence were integrated into the database structure. A core data set of 35 variables for which data collection was considered mandatory was established prior to data collection in the registry. Data monitoring was performed by a central administrator. All data were prospectively collected in the Traumabase registry except admission myoglobin, CK and phosphate that were retrospectively acquired.

All patients had a contrast-enhanced whole-body computed tomography (CT) to assess injury severity in the initial phase of care. In the database, hemorrhagic shock was defined by transfusion requirements of at least four units of packed red blood cells (RBC) within the first 6 h [18, 19].

### Renal function assessment

Acute kidney injury (AKI) was assessed according to the Kidney Disease Improving Global Outcomes (KDIGO) [20] classification from maximum creatinine over the first 7 days of ICU stay. Baseline creatinine was not available for trauma patients. As stated in the KDIGO guidelines, we back-calculated baseline creatinine according to the Modification of Diet in Renal Disease (MDRD) formula with a glomerular filtration rate (GFR) of 75 mL/min per 1.73 m^2^. To be consistent with the two established predictive models that we used in the study, we also considered two other definitions of baseline creatinine: admission creatinine [21] and the lowest creatinine over the first 5 days of admission or until discharge whichever came first [4].

### Study endpoints

The study primary endpoint was AKI (any stage of the KDIGO classification) over the first admission week. The secondary endpoints were AKI stage 2 or 3 and the occurrence of severe rhabdomyolysis as defined by a peak of myoglobin higher than 5000 μg/L as stated in several studies [22, 23] or a peak of CK higher than 5000 U/L as proposed in the statement on prevention and management of acute renal failure in the ICU” published in 2010 [24] over the first 7 days of admission.

### Statistical analysis

Sample size calculation was done according to the method described by Obuchowski et al. [25]. To show a difference of 0.08 in the AUC–ROC of myoglobin and CK to predict AKI with a power of 90% and *α* = 0.05, assuming that the prevalence of AKI any stage was 12% [4], we calculated that at least 825 patients should be included in the study. Details regarding sample size calculation are given in Additional file 2.

Quantitative variables were expressed as mean (SD) or median [25th–75th interquartiles] according to their distribution and categorical variables were expressed as count (proportion). Comparison of two Gaussian variables was performed with a Student *t* test, comparison of non-normally distributed variables was done with the Mann Whitney test and proportions were compared with the Chi-square test.

A simple linear regression model was used to assess the correlation between two variables. We built receiver operating characteristics curves for various thresholds of admission myoglobin and admission CK to predict AKI or severe rhabdomyolysis. Comparison of two ROC curves for AKI prediction was achieved by undergoing a non-parametric approach (bootstrap method with 2000 replicates) [26]. The best threshold was defined as the value maximizing the Youden index (sensitivity + specificity − 1).

To further assess the association of admission CK and myoglobin with AKI, we forced both variables into two prediction models of AKI that were recently reported in two different cohorts of trauma patients [4, 21]. For each model, when myoglobin or CK coefficient was statistically significant, we calculated the continuous net reclassification index (NRI) and the integrated discrimination improvement (IDI) to assess their ability to reclassify AKI patients. Model calibration was assessed using the Hosmer–Lemeshow statistical test and model discrimination was assessed by calculation of the AUC–ROC. Decision curve analysis was used to assess the net benefit of CK, myoglobin and multivariable models with AKI prediction [27, 28]. We conducted a multiple imputation of missing data for the variables used in the two predictive models (MICE package, R) [29] except AKI status, admission CK and myoglobin that were complete. Additional details are provided in Additional file 3 regarding the multivariable analysis.

This analysis followed the TRIPOD (Transparent Reporting of a multivariable prediction model for Individual Prognosis Or Diagnosis) recommendations for prediction models [30] and standards for reporting diagnostic accuracy (Additional file 4).

Two-sided level of significance was fixed at 5%. Results were analyzed using R open source software 3.4.1 (http://www.R-project.org) and Prism (Graphpad Software, San Diego, USA).

## Results

### General characteristics of the study population

**Fig. 1:**
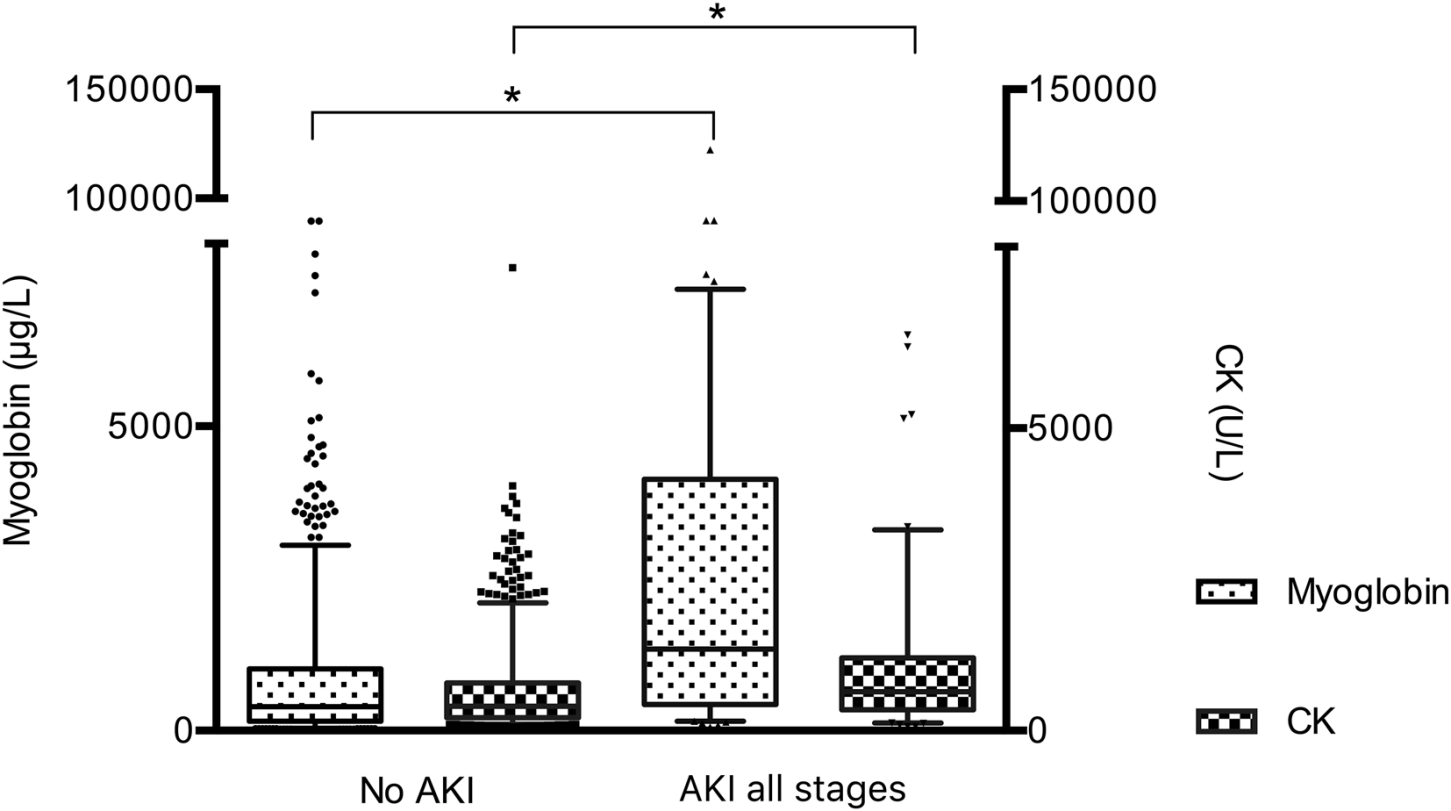
Admission CK and myoglobin in patients with and those without AKI. *p < 0.001. *AKI* Acute Kidney Injury, *CK* creatine kinase

Of the 1325 patients admitted to our centre during the study period, 857 met the inclusion criteria (flow chart is available in Additional file 5). Patients’ characteristics are reported in Table 1. Trauma patients were predominantly middle-aged men with a median ISS of 17 [9–29]. Hemorrhagic shock occurred in 7% of patients. Overall mortality was 12.1%. 102 patients (11.9%) experienced AKI (any stage). 69 (8.1%) had KDIGO 1, 12 (1.4%) had KDIGO 2 and 21 (2.5%) had KDIGO 3 AKI. 9 patients required renal replacement therapy during ICU stay. A comparison between AKI and non-AKI patients is provided in Table 1. Both admission values for CK and for myoglobin were higher in AKI patients than in non-AKI patients (CK: 643 [350–1185] U/L and 401 [211–780] U/L, respectively, *p* < 0.001; myoglobin: 2738 [595–7286] µg/L and 382 [167–1245] µg/L, respectively, *p* < 0.001), Fig. 1. There was only a very weak correlation between admission myoglobin and CK (*R*^2^ = 0.19, *p* < 0.001) and also between maximum myoglobin and maximum CK (*R*^2^ = 0.34, *p* < 0.001) suggesting that both biomarkers are not interchangeable (Additional file 6). Severity of Injury defined by ISS was clearly associated with higher admission myoglobin levels (Additional file 7).

**Table 1:** General characteristics of the study population according to AKI status

Characteristics	Whole population	No AKI	AKI all stages	*p*
	*n* = 857	*n* = 755	*n* = 102	
General characteristics				
Age, year	36 [25–52]	34 [25–49]	56 [36–67]	< 0.001
Ratio m/f, *n* (%)	667/190 (78/22)	577/178 (76/24)	90/12 (88/12)	0.007
SAPS II	20 [12–47]	18 [11–41]	57 [50–64]	< 0.001
SOFA 24 h	2 [0–9]	1 [0–6]	12 [8–14]	< 0.001
ISS	17 [9–29]	14 [9–26]	34 [24–50]	< 0.001
Blunt/penetrating trauma, *n* (%)	821/36 (96/4)	721/34 (95/5)	100/2 (98/2)	0.348
Prehospital time, min	94 [75–120]	92 [75–115]	105 [85–136]	0.003
Predicted mortality (TRISS), %	15.9	11.8	46.7	–
Prehospital characteristics				
GCS	15 [12–15]	15 [14–15]	12 [4–15]	< 0.001
Minimum SBP, mmHg	114 [99–129]	117 [100–130]	92 [73–117]	< 0.001
Maximum HR, bpm	94 [81–107]	94 [82–106]	98 [71–123]	0.406
Minimum SpO_2_, %	97 [95–99]	97 [95–99]	96 [91–98]	< 0.001
Crystalloids, mL	500 [250–1000]	500 [250–750]	750 [500–1500]	< 0.001
Colloids, mL	0 [0–0]	0 [0–0]	0 [0–0]	< 0.001
Use of vasopressors, *n* (%)	106 (12.4)	66 (8.8)	40 (39.6)	< 0.001
Hospital admission				
SBP, mmHg	127 [113–142]	128 [115–143]	120 [96–138]	0.002
pH	7.36 [7.30–7.41]	7.37 [7.32–7.41]	7.28 [7.20–7.35]	< 0.001
Lactate, mmol/L	1.9 [1.2–2.8]	1.7 [1.1–2.5]	3.2 [2.1–5.3]	< 0.001
Hemoglobine, g/dL	13.4 [11.9–14.4]	13.5 [12.3–14.5]	11.4 [9.4–12.9]	< 0.001
Fibrinogen, g/L	2.3 [2–2.8]	2.4 [2–2.8]	2.0 [1.3–2.6]	< 0.001
Creatinine, μmol/L	78 [66–93]	76 [65–87]	115 [91–137]	< 0.001
Phosphate, mmol/L	0.92 [0.74–1.14]	0.89 [0.72–1.09]	1.26 [0.98–1.76]	< 0.001
Myoglobin on admission, μg/L	447 [177–1149]	393 [154–1015]	1332 [427–4074]	< 0.001
Maximum myoglobin, μg/L	552 [196–1808]	482 [167–1245]	2738 [595–7286]	< 0.001
CK on admission, U/L	423 [215–832]	401 [211–780]	643 [350–1185]	< 0.001
Maximum CK, U/L	810 [283–2484]	757 [268–2036]	2530 [485–7441]	< 0.001
Transfusion				
Hemorrhagic shock, *n* (%)	60 (7)	36 (4.8)	24 (23.5)	< 0.001
Outcomes during hospital stay				
ICU length of stay, days	4 [2–8]	4 [2–7]	7 [3–16]	< 0.001
Mortality, *n* (%)	103 (12.1)	62 (8.2)	41 (40.2)	< 0.001

Data are given as median [interquartile range] or number (proportions)

*CK* creatine kinase, *GCS* Glasgow Coma Scale, *HR* Heart Rate, *ICU* intensive care unit, *ISS* injury severity score, *SBP* systolic blood pressure, *SAPS* Simplified Acute Physiology Score, *SOFA* sequential organ failure assessment score, *SpO*_*2*_ pulse oximeter oxygen saturation, *TRISS* Trauma and Injury Severity Score

*p* is given for comparison between patients without AKI and those with AKI all stages

### Prediction of AKI

**Fig. 2:**
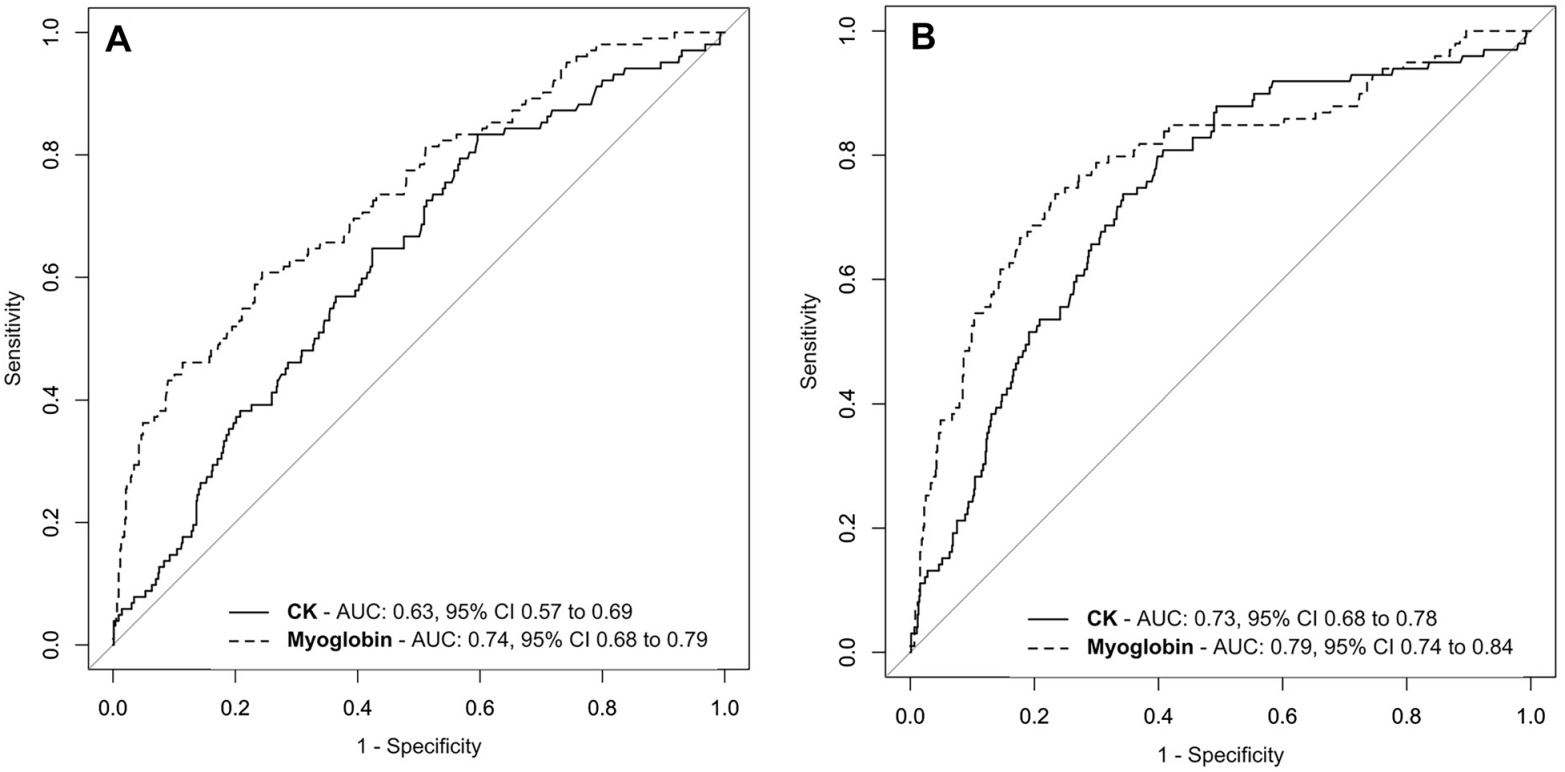
Receiver operating characteristics curves of admission CK and myoglobin for prediction of AKI any stage (**A**) or AKI stage 2 or 3 (**B**). The thin bisector line is the non-discrimination line. *AUC* area under the curve, *CI* confidence interval, *CK* creatine kinase

Admission myoglobin performed better than CK for prediction of AKI any stage, (AUC–ROC 0.74 95% CI 0.68–0.79 and 0.63 95% CI 0.57–0.69, respectively, *p* < 0.001) as well as for prediction of AKI KDIGO 2 or 3 (AUC–ROC 0.79 95% CI 0.74–0.84 and 0.74 95% CI 0.69–0.79, respectively, *p* = 0.002) (Fig. 2). Admission myoglobin had a 61% sensitivity and 76% specificity to predict AKI any stage, whereas it had a 74% sensitivity and 77% specificity to predict AKI stage 2 or 3. Further performances of admission myoglobin and CK are provided in Additional file 8.

When individually entered in 6 multivariable models (i.e., 2 validated models, each one applied to 3 AKI definitions) predicting post-traumatic AKI, admission myoglobin had an odds ratio that was significantly greater than 1 in five models (Table 2). Admission CK odds ratio was significantly greater than 1 in 2 models. When admission myoglobin and CK were simultaneously entered in the models, admission myoglobin was retained in the final model, while CK was not, except in one model, where both were retained (data not shown). Assessment of model calibrations is provided in Additional file 9, showing results of the Hosmer–Lemeshow tests conducted for each model. The most effective model was the one including age, admission phosphate, hemorrhagic shock, admission creatinine and admission myoglobin. Further performances of each variable in both models are provided in Additional files 10 and 11.

**Table 2:** Added predictive value provided by admission CK and myoglobin for AKI prediction

	Model 1			Model 2		
	Base	Myoglobin	CK	Base	Myoglobin	CK
Estimation of baseline creatinine with MDRD formula						
OR (95% CI)	–	1.017 (1.003–1.034)	1.007 (0.999–1.042)	–	1.026 (1.011–1.041)	1.002 (0.999–1.028)
*p*-value OR	–	0.046	0.687	–	< 0.001	0.866
AUC (95% CI)	0.897 (0.861–0.931)	0.902 (0.862–0.935)	0.897 (0.860–0.931)	0.798 (0.744–0.847)	0.805 (0.752–0.856)	0.799 (0.745–0.850)
Estimation of baseline creatinine with creatinine on admission						
OR (95% CI)	–	1.017 (1.003–1.032)	1.019 (0.999–1.043)	–	1.011 (0.999–1.025)	0.999 (0.998–1.002)
*p*-value OR	–	0.019	0.137	–	0.15	0.717
AUC (95% CI)	0.745 (0.691–0.794)	0.759 (0.705–0.808)	0.746 (0.690–0.801)	0.767 (0.714–0.811)	0.769 (0.718–0.815)	0.766 (0.745–0.837)
Estimation of baseline creatinine with lowest creatinine over the first 5 days						
OR (95% CI)	–	1.050 (1.035–1.065)	1.063 (1.039–1.086)	–	1.034 (1.019–1.050)	1.033 (1.010–1.056)
*p*-value OR	–	< 0.001	< 0.001	–	< 0.001	0.006
AUC (95% CI)	0.654 (0.616–0.694)	0.773 (0.739–0.806)	0.681 (0.642–0.718)	0.782(0.748–0.816)	0.798 (0.764–0.830)	0.787 (0.758–0.822)

Base model 1 is the model established by Haines et al. [21] that includes age, admission phosphate, admission creatinine and hemorrhagic shock as independent variables. Base model 2 is the model established by Harrois et al. [4] that includes maximum prehospital heart rate, minimum systolic blood pressure, admission lactate, injury severity score (ISS) and hemorrhagic shock. Models were constructed for 3 AKI definitions that differ according to baseline creatinine definition. Myoglobin and CK were forced into each model. AUC are given for each model. OR are given per 100 μg/L for myoglobin and per 100 U/L for CK. NRI was calculated only when OR for myoglobin or CK was significantly higher than 1

*AUC* area under the receiver operating characteristic curve, *CK* creatine kinase, *NRI* net reclassification index, *OR* odds ratio

Using reclassification methods, the addition of myoglobin in clinical models resulted in a statistically significant improvement in AKI prediction: NRI was constantly greater than 0 (5 models over 5) ranging from 0.201 (0.001–0.403) to 0.381 (0.176–0.586) and IDI was greater than 0 in 3 over 5 models ranging from 0.008 (− 0.003–0.018) to 0.039 (0.015–0.064). The addition of CK in clinical models never resulted in a significant ability to reclassify patients for AKI risk (Additional file 12).

**Fig. 3:**
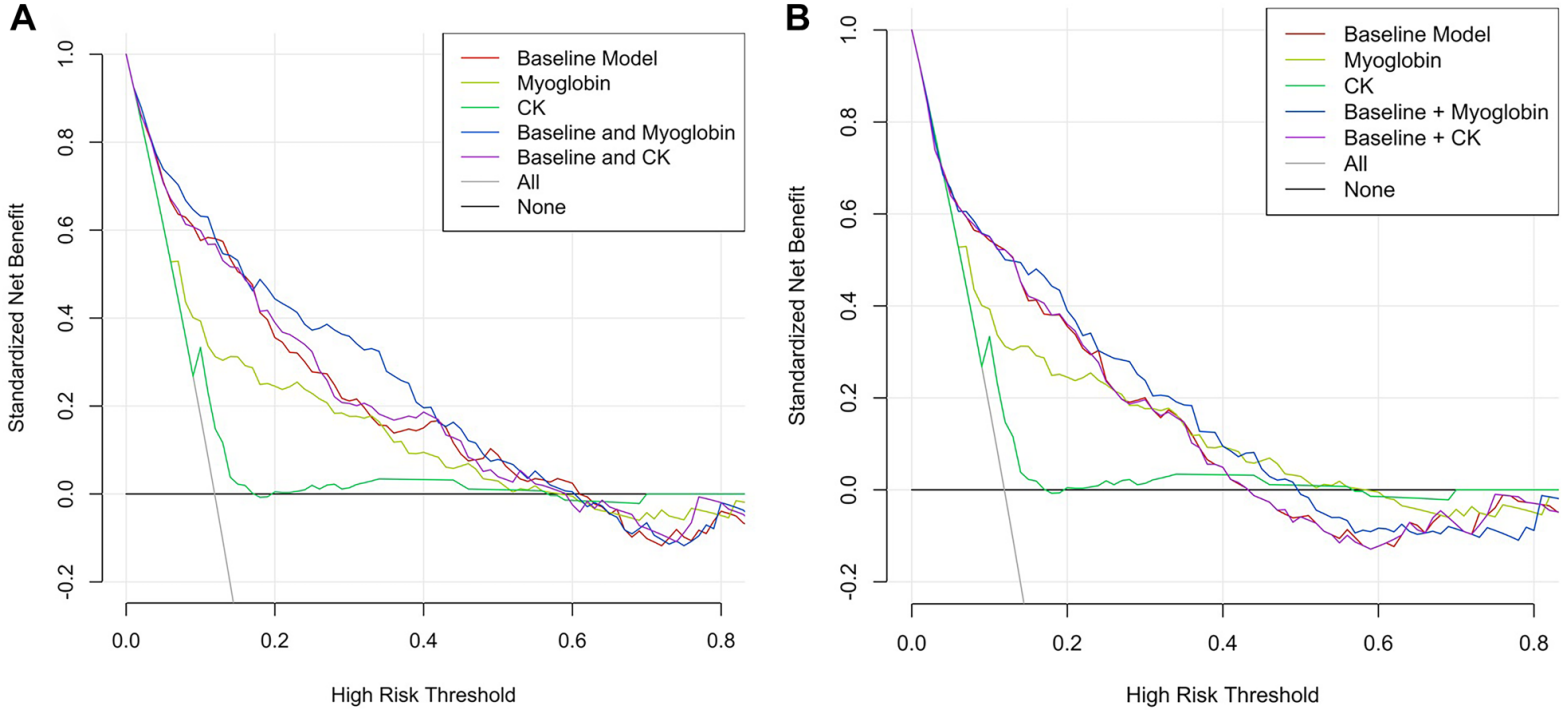
Decision curve showing the performance of CK and myoglobin alone or forced in 2 established predictive models of post-traumatic AKI: **A** model established by Haines et al. in which myoglobin showed a net benefit in range 5–11% + 17–40%, **B** model established by Harrois et al. in which myoglobin showed a net benefit in range 15–45%. The line “all” corresponds to the situation, where all patients will develop AKI, while “None” is obtained by considering that no patient will develop AKI. A model has net benefit when it has greater benefit than the 2 basic lines (“all” and “none”) or when it shows greater benefit than a given model

Using decision curve analysis, the addition of myoglobin predicted AKI with net benefit in range (5–11% + 17–40%, Fig. 3A) and (15–45%, Fig. 3B) in the first and second validated model, respectively, while CK did not add net benefit.

### Prediction of severe rhabdomyolysis

64 patients (7.5%) had maximum myoglobin exceeding 5000 μg/L, whereas 113 patients (13.2%) had maximum CK exceeding 5000 U/L. Performances of admission myoglobin and CK to predict severe rhabdomyolysis are reported in Table 3. Admission myoglobin performed significantly better than admission CK to predict severe rhabdomyolysis either with a maximum myoglobin exceeding 5000 μg/L (AUC–ROC 0.94 95% CI (0.91–0.97) and 0.88 95% CI (0.84–0.91), respectively, *p* < 0.001) or a maximum CK exceeding 5000 U/L (AUC–ROC 0.91 95% CI (0.88–0.94) and 0.88 95% CI (0.85–0.91), *p* = 0.013). The optimal cutoff for admission myoglobin to predict severe rhabdomyolysis defined by maximum myoglobin exceeding 5000 μg/L was 1938 μg/L, while it was 1193 μg/L to predict maximum CK exceeding 5000 U/L.

**Table 3:** Performances of admission myoglobin and CK to predict severe rhabdomyolysis

Performance parameter	Prediction of severe rhabdomyolysis defined by a maximum myoglobin > 5000 μg/L		Prediction of severe rhabdomyolysis defined by a maximum CK > 5000 U/L	
	Admission myoglobin	Admission CK	Admission myoglobin	Admission CK
AUC-ROC	0.94 (0.91–0.97)*	0.88 (0.84–0.91)*	0.91 (0.88–0.94)^#^	0.88 (0.85–0.91)^#^
Optimal cutoff	1938	850	1193	686
Sensitivity	84 (74–91)	83 (72–90)	85 (77–90)	88 (81–93)
Specificity	90 (88–92)	80 (77–83)	84 (81–87)	75 (72–78)
PPV	41 (33–49)	25 (20–32)	45 (39–52)	35 (30–41)
NPV	99 (97–99)	98 (97–99)	97 (96–98)	98 (96–99)
PLR	8.5 (6.7–10.7)	4.2 (3.5–5.0)	5.4 (4.5–6.5)	3.6 (3.1–4.1)
NLR	0.2 (0.1–0.3)	0.2 (0.1–0.4)	0.2 (0.1–0.3)	0.2 (0.1–0.3)

Severe rhabdomyolysis was defined either by myoglobin > 5000 μg/L or CK > 5000 U/L. Performance parameters are given with their 95% confidence interval

*AUC–ROC* area under the receiver operating characteristic curve, *NLR* negative likelihood ratio, *NPV* negative predictive value, *PLR* positive likelihood ratio, *PPV* positive predictive value

*p < 0.001, ^#^*p* = 0.013 for comparison between AUC–ROC of admission myoglobin and that of admission CK

## Discussion

We aimed to compare the ability of admission myoglobin and CK to predict AKI and severe rhabdomyolysis in trauma patients. First, we found that admission myoglobin better predicted AKI than admission CK in trauma patients. Second, admission myoglobin added significant information to established multivariable models of post-traumatic AKI prediction, while admission CK marginally contributed to improve these models. Third, admission myoglobin was a better predictor of severe rhabdomyolysis than admission CK.

### Relationship with previous studies

Several studies have shown myoglobin and CK concentrations to be associated with the occurrence of AKI in the setting of rhabdomyolysis. However, most of them focused on the association between CK or myoglobin peaks [3, 4, 7, 9, 10, 31] and AKI. Of note, a few of them compared CK and myoglobin peaks to predict AKI and reported myoglobin to perform better [8, 9, 22, 23]. Though CK and myoglobin peaks relate to the extent of intramuscular content release and, therefore, are surrogate of rhabdomyolysis severity, they occur late in the course of rhabdomyolysis [8]. This latter point makes them irrelevant to predict post-traumatic AKI that commonly occurs within 48 h after trauma [4].

A few studies focused on CK and myoglobin measured on admission. They mostly included patients with non-traumatic rhabdomyolysis and reported that both muscular proteins were found in greater proportion in patients who later developed AKI than those who did not [11, 13, 14, 32]. However, none of these studies compared the abilities of both markers to predict AKI. In this study, we found that admission myoglobin better predicted AKI than admission CK. Moreover, CK was not retained as a significant predictor of AKI in multivariable analysis, while myoglobin was. Decision curve analysis showed improved net benefit in ranges from 10 to 40% for AKI classification with models including myoglobin. AKI prevention is based on measures that do not cause to much potential harm to patients (hemodynamic optimisation and avoidance of nephrotoxic drugs), which is a setting corresponding to a mild classification threshold of 10 to 40%. We consider these results of importance, since CK and myoglobin are both regularly measured in the setting of rhabdomyolysis and choosing one over the other may thus lead to better AKI prediction. In a study done by Haines et al., admission CK was already not retained in their multivariable model predicting AKI after trauma [21]. In contrast McMahon et al. reported that admission CK higher than 40,000 U/L was an independent predictor of hospital mortality or renal replacement therapy in patients admitted to hospital for rhabdomyolysis [12]. However, more than a third of patients had causes of rhabdomyolysis that potentially started long before admission (i.e., sepsis, immobilisation). This likely resulted in high CK values on admission linked to prolonged and advanced rhabdomyolysis, suggesting these patients were close to reaching CK peak. In contrast, no patient had CK above 8000 U/L on admission in our study and time from trauma to admission was short suggesting that CK and myoglobin were measured at the very beginning of the rhabdomyolysis process.

There are several hypotheses to explain the ability of admission myoglobin to better predict AKI than admission CK. First, free myoglobin is a direct mediator of rhabdomyolysis-induced AKI [6, 33–35], while CK has no reported effect on kidney. Thus, admission myoglobin may more accurately reflect the renal toxicity of rhabdomyolysis than admission CK. This is further supported by the better ability of admission myoglobin to predict severe rhabdomyolysis than CK. Second, unlike CK, myoglobin release does not depend on lymphatic transport and is faster released into the bloodstream, which makes it more accurate as an early marker for risk stratification of rhabdomyolysis-induced AKI [36, 37]. This also may be a reason explaining why myoglobin and CK are not interchangeable markers of rhabdomyolysis. However, on top of its direct renal toxicity, one might speculate that myoglobin also reflects the extent of tissue injury leading to systemic inflammation that ultimately causes renal function impairment.

### Implications of study findings

Our findings imply that admission myoglobin should be preferred over CK to predict AKI in trauma patients. Measurement of myoglobin is part of the admission blood test done on arrival to the hospital and is routinely available in biochemistry labs. This study also implies that myoglobin provides additional predictive value to established models of AKI prediction following major trauma, while CK does not. Hence, admission myoglobin should be added to these models to achieve better post-traumatic AKI prediction. In addition, unlike what is regularly stated, we think this study also supplies arguments to use myoglobin rather than CK to assess rhabdomyolysis severity in trauma patients [38, 39]. Moreover, the results of this study suggest the following clinical message: an admission myoglobin greater than 1200 µg/L predicts not only AKI (KDIGO 2 or 3) with sensitivity and specificity of 74 and 77%, respectively, but also severe rhabdomyolysis (occurrence of a CK peak greater than 5000 U/L) with sensitivity and specificity of 84 and 90%, respectively. Finally, in a trauma setting, predicting the severity of rhabdomyolysis at the peak based on clinical injuries is challenging (whether a patient trapped in a vehicle was really crushed or not for instance). Admission myoglobin provides information to assess the risk for developing severe rhabdomyolysis following crush injuries and may help decide to start (or not) early preventive actions, such as intensive hydration [24], to decrease AKI risk.

### Strengths and limitations

This study has several strengths. This is the largest study reporting measurement of admission myoglobin and CK in severe trauma patients. Moreover, our results apply to a wide range of trauma patients as we did not select them according to a predefined maximum CK threshold. In addition, our results are consistent whatever the definition used to assess baseline creatinine.

We acknowledge several limitations. First, this is a retrospective, single-centre study. However, most of the data used in the study were prospectively collected and registered in a research database. Second, this study exclusively included trauma patients; therefore, results cannot be extended to other causes of rhabdomyolysis. Third, optimal cutoffs are given for patients spending a median amount of time of 94 min in the prehospital setting, thus, these thresholds may not apply to trauma systems with different prehospital times. Fourth, AKI was assessed from maximum creatinine only. By not considering urine output, we may have underestimated AKI occurrence. Fifth, the use of nephrotoxic agents (amount of IV contrast received, antibiotics and regular angiotensin converting enzyme inhibitors) would have added relevant information to the study but these data are not collected in our database. Sixth, we acknowledge that no baseline creatinine was available in the study cohort. Consequently, we had to estimate baseline creatinine, which may introduce bias in AKI assessment. However, we used the three more commonly used methods to estimate baseline creatinine to evaluate whether our results were consistent whatever the definition used.

## Conclusions

Admission myoglobin better predicts the development of AKI after major trauma, and should be added in established predictive models of post-traumatic AKI to early identify high-risk patients. These results also suggest that myoglobin should be preferred over CK to assess rhabdomyolysis severity.

## Supplementary Information

**Additional file 1.** The Vittel algorithm.

**Additional file 2.** Sample size calculation.

**Additional file 3.** Multivariable logistic regression models for AKI prediction.

**Additional file 4.** Standard for reporting diagnostic accuracy (STARD) checklist.

**Additional file 5.** Linear regression between CK and myoglobin.

**Additional file 6.** Flow chart.

**Additional file 7.** Median and mean admission myoglobin levels according to ISS quartile.

**Additional file 8.** Predictive performances of initial myoglobin and initial CK for acute kidney injury (KDIGO any stage) with baseline creatinine calculated with MDRD formula.

**Additional file 9.** Calibration of multivariable models as assessed by the Hosmer–Lemeshow statistical test.

**Additional file 10.** Performances of variable from multivariable models to predict acute kidney injury (KDIGO any stage).

**Additional file 11.** Performances of variable from multivariable models to predict acute kidney injury (KDIGO stage 2 or 3).

**Additional file 12.** Continuous net reclassification improvement (NRI) and IDI when adding admission myoglobin and CK to 2 established models of post-traumatic AKI prediction.

## Data Availability

The data sets supporting the conclusions of this article are available from the corresponding author on reasonable request.

## Abbreviations

AIS: Abbreviated injury scale
AKI: Acute kidney injury
AUC–ROC: Area under the ROC curve
CCTIRS: Advisory Committee for Information Processing in Health Research
CERAR: Research ethics committee of the French society of anesthesia and intensive care
CK: Creatine kinase
CNIL: Advisory committee for information processing in health research
CT: Computed tomography
GFR: Glomerular filtration rate
ICU: Intensive care unit
IDI: Integrated discrimination index
ISS: Injury severity score
IV: Intravenous
KDIGO: Kidney disease improving global outcome
MDRD: Modification of diet in renal disease
MICE: Multivariate imputation by chained equations
NRI: Net reclassification index
RBC: Red blood cell
ROC: Receiver operating characteristics
SAPS: Simplified acute physiology score
SD: Standard deviation
SOFA: Sequential organ failure assessment
TRIPOD: Transparent reporting of a multivariable prediction model for individual prognosis or diagnosis
TRISS: Trauma injury severity score
