# Supplementary material for: Admission serum myoglobin and the development of acute kidney injury after major trauma
Source: Ann Intensive Care. 2021 Sep 24;11:140. doi: 10.1186/s13613-021-00924-3 (PMC8463647; doi:10.1186/s13613-021-00924-3)
Supplement: Supplementary file 1 — Additional file 1. The Vittel algorithm. [file 13613_2021_924_MOESM1_ESM.docx]

**Additional file 1:** Vittel algorithm

The Vittel algorithm is applied from step 1 to 5. Presence of one criterium suggests to transport patients to a level one trauma centre except step 5 for which a decision is made on a case by case basis. The Vittel criteria were introduced by the « Société Française de Médecine d’Urgence (SFMU) » in 2002. GCS = Glasgow Coma Scale, SpO_2_ = pulse oximeter oxygen saturation, SBP = Systolic blood pressure.

Step one (physiological signs)

Step four (resuscitation)

Age > 65

Cardiac insufficiency, respiratory failure or ischemic heart disease

Pregnancy (2d, 3d trimester)

Coagulation disorders

Step five (medical history)

Mechanical ventilation

Volume load > 1000 mL

Vasopressor

Shock trousers

Penetrating trauma of head, neck, thorax, abdomen

Flail chest

Severe burn

Pelvic fracture

Suspicion of medullar injury

Amputation at or above wrist or ankle level

Acute limb ischemia

Step three (anatomical injuries)

Ejection from vehicle

Death in same passenger compartment

Fall > 6m

Victim thrown or projected

Global assessement of speed and potential injuries:

Vehicle deformation, estimated vehicle speed, helmet absent, seat belt not fastened

Blast

Step two (global assessment of speed and mechanism)

GCS < 13 and/or

SBP < 90 mmHg and/or

SpO2 < 90%
