# Supplementary material for: Admission serum myoglobin and the development of acute kidney injury after major trauma
Source: Ann Intensive Care. 2021 Sep 24;11:140. doi: 10.1186/s13613-021-00924-3 (PMC8463647; doi:10.1186/s13613-021-00924-3)
Supplement: Supplementary file 2 — Additional file 2. Sample size calculation. [file 13613_2021_924_MOESM2_ESM.docx]

**Additional file 2 :** Sample size calculation

Sample size calculation was done according to the method described by Obuchowski et al ^1^ by using power.roc.test function in R pROC package ^2^. Since no empirical ROC curve is known for AKI prediction either by initial myoglobin or initial CK level in trauma patients, we used data from a previous prospective study that included 40 trauma patients with concomitant measurements of initial myoglobin and CK (communication, Congress of the French Society of Anaesthesiology and Intensive Care 2017 (Société Française d’Anesthésie-Réanimation)). From this small sample study, we extracted binormal parameters A and B for the ability of myoglobin and CK to predict AKI (any stage of KDIGO classification) (where A=(μ_AKI_ - μ_no AKI_)/σ_AKI_ and B=σ_no AKI_/σ_AKI_ with μ and σ being the mean and standard deviation of the measured variables (myoglobin and CK) in the AKI and non AKI group) (see Table below). The correlation between myoglobin and CK variables were also calculated for patients with AKI (rA) and patient without AKI (rN) (see Table below). In a second study done by our group, prevalence of AKI any stage was 12% ^3^ (lower limit of CI) in patients having CK/myoglobin measurement on arrival at hospital. To show a difference of 0.08 in the AUC-ROC of myoglobin and CK to predict AKI with a power of 90% and α=0.05, we calculated that at least 825 patients should be included in the study (see R script below).

**Table describing Binormal parameters for the ability of myoglobin and CK to predict AKI**

|  | **A** | **B** | **rA** | **rN** | **Kappa** |
| --- | --- | --- | --- | --- | --- |
| **CK** | 0.39 | 0.23 | 0.78 | 0.92 | Ratio controls over cases (1/0.12=8) |
| **Myoglobin** | 0.6 | 0.37 |  |  |  |

**R script used to calculate the sample-size**

Library(pROC)

ob.param<-list(A1=0.60, B1=0.37, A2=0.39, B2=0.23, rn=0.92, ra=0.78,delta=0.08)

power.roc.test(ob.param, sig.level=0.05, power=0.9, alternative="two.sided", kappa=8, ncases=NULL, ncontrols=NULL)
