# Supplementary material for: Admission serum myoglobin and the development of acute kidney injury after major trauma
Source: Ann Intensive Care. 2021 Sep 24;11:140. doi: 10.1186/s13613-021-00924-3 (PMC8463647; doi:10.1186/s13613-021-00924-3)
Supplement: Supplementary file 3 — Additional file 3. Multivariable logistic regression models for AKI prediction. [file 13613_2021_924_MOESM3_ESM.docx]

**Additional file 3:** Multivariable logistic regression models for AKI prediction

In order to further assess the predictive value of initial CK and myoglobin to predict AKI, we forced both variables in two prediction models of AKI that were recently constructed in two different cohorts of trauma patients ^3,4^. As reported by the authors, we used age, admission phosphate, admission creatinine and presence of hemorrhagic shock as predictors in the first model ^4^. The Charlson index could not be calculated and was not included in the model (see Table below). We used ISS, hemorrhagic shock, lactate, maximal prehospital heart rate and minimum prehospital mean arterial pressure as predictors in the second model ^3^. We did not include the variable “direct transfer to trauma centre” since all patients were directly admitted from the scene (see Table below). First, we constructed two logistic regression models with the above-mentioned variables to assess the ability of both established models to predict AKI in the study cohort (see Table below). The occurrence of AKI stage 1 to 3 (according to baseline creatinine back-calculated with MDRD formula) was the dependent variable. Then, initial CK and myoglobin were individually forced in both models. The model calibration was assessed by using the Hosmer-Lemeshow statistic and the discrimination by reporting the area under the receiver operating characteristic (AUC-ROC) curve. We conducted the same analysis with two other definitions of baseline creatinine (creatinine on admission as reported in the study that established the first model ^4^ and the lowest creatinine over the first 5 days of ICU as reported in the study that established the second model ^3^), making up two additional models for each definition (see Table below). When the model coefficient of myoglobin or CK was statistically significant, we calculated the continuous net reclassification index (NRI) to assess their reclassification ability.

**Table describing dependent variables and AKI definitions used in the predictive models**

|  | **Original study** | **Present study** |
| --- | --- | --- |
| **Model 1 established by Haines et al.** ^4^ | | |
| **Independent variables** | Age | Age |
|  | Packed red blood cells transfused in first 24h | Packed red blood cells transfused in first 6h greater than or equal to 4 (yes/no) |
|  | Admission phosphate | Admission phosphate |
|  | Admission creatinine | Admission creatinine |
|  | Charlson comorbidity index | - |
| **AKI definition** | AKI stage 1, 2 or 3 of KDIGO classification with admission creatinine as baseline creatinine | AKI stage 1, 2 or 3 of KDIGO classification with 3 different definitions of baseline creatinine:   - admission creatinine - creatinine back-calculated with MDRD formula (GFR=75 mL/min) - lowest creatinine over the first 5 days |
| **Model 2 established by Harrois et al.** ^3^ | | |
| **Independent variables** | Injury Severity Score (ISS) | Injury Severity Score (ISS) |
|  | Packed red blood cells transfused in first 6h greater than or equal to 4 (yes/no) | Packed red blood cells transfused in first 6h greater than or equal to 4 (yes/no) |
|  | Admission lactate | Admission lactate |
|  | Maximum prehospital HR | Maximum prehospital HR |
|  | Minimum prehospital MAP | Minimum prehospital MAP |
|  | Direct transfer to trauma centre (yes/no) | - |
| **AKI definition** | AKI stage 1, 2 or 3 of KDIGO classification with the lowest creatinine over the first five days of admission as baseline creatinine | AKI stage 1, 2 or 3 of KDIGO classification with 3 different definitions of baseline creatinine:   - admission creatinine - creatinine back-calculated with MDRD formula (GFR=75 mL/min) - lowest creatinine over the first 5 days |

1. Obuchowski NA, McClish DK: Sample size determination for diagnostic accuracy studies involving binormal ROC curve indices. Stat Med 1997; 16:1529–42

2. Robin X, Turck N, Hainard A, Tiberti N, Lisacek F, Sanchez J-C, Müller M: pROC: an open-source package for R and S+ to analyze and compare ROC curves. BMC Bioinformatics 2011; 12

3. Harrois A, Soyer B, Gauss T, Hamada S, Raux M, Duranteau J, Traumabase® Group: Prevalence and risk factors for acute kidney injury among trauma patients: a multicenter cohort study. Crit Care 2018; 22:344

4. Haines RW, Lin S-P, Hewson R, Kirwan CJ, Torrance HD, O’Dwyer MJ, West A, Brohi K, Pearse RM, Zolfaghari P, Prowle JR: Acute Kidney Injury in Trauma Patients Admitted to Critical Care: Development and Validation of a Diagnostic Prediction Model. Sci Rep 2018; 8:3665
