# Supplementary material for: Admission serum myoglobin and the development of acute kidney injury after major trauma
Source: Ann Intensive Care. 2021 Sep 24;11:140. doi: 10.1186/s13613-021-00924-3 (PMC8463647; doi:10.1186/s13613-021-00924-3)
Supplement: Supplementary file 5 — Additional file 5. Linear regression between CK and myoglobin. [file 13613_2021_924_MOESM5_ESM.docx]

12 had no creatinine measure

84 had no CK or myoglobin measure

11 were < 18 years

361 were not directly admitted to trauma centre

857 patients included in the analysis

953 patients ≥ 18 and directly admitted to trauma centre

1325 patients were admitted between January 2015 and June 2017
