# Supplementary material for: Admission serum myoglobin and the development of acute kidney injury after major trauma
Source: Ann Intensive Care. 2021 Sep 24;11:140. doi: 10.1186/s13613-021-00924-3 (PMC8463647; doi:10.1186/s13613-021-00924-3)
Supplement: Supplementary file 6 — Additional file 6. Flow chart. [file 13613_2021_924_MOESM6_ESM.docx]

**Additional file 6:** Linear regression between CK and myoglobin


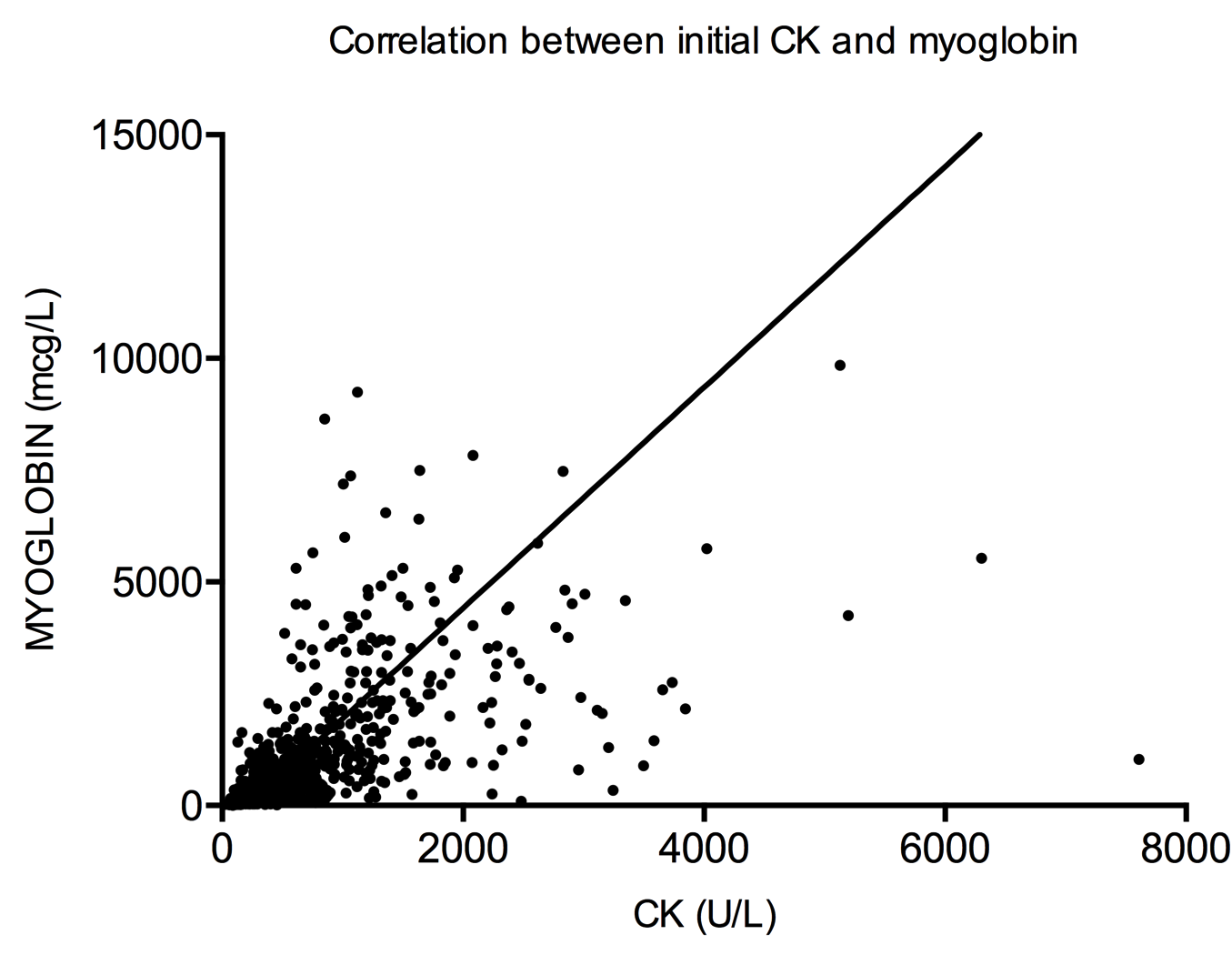


Slope=2.46, R^2^=0.19, p<0.001. 2 outliers are not reported in the graph because of myoglobin levels of 17000 and 121000 µg/L respectively.


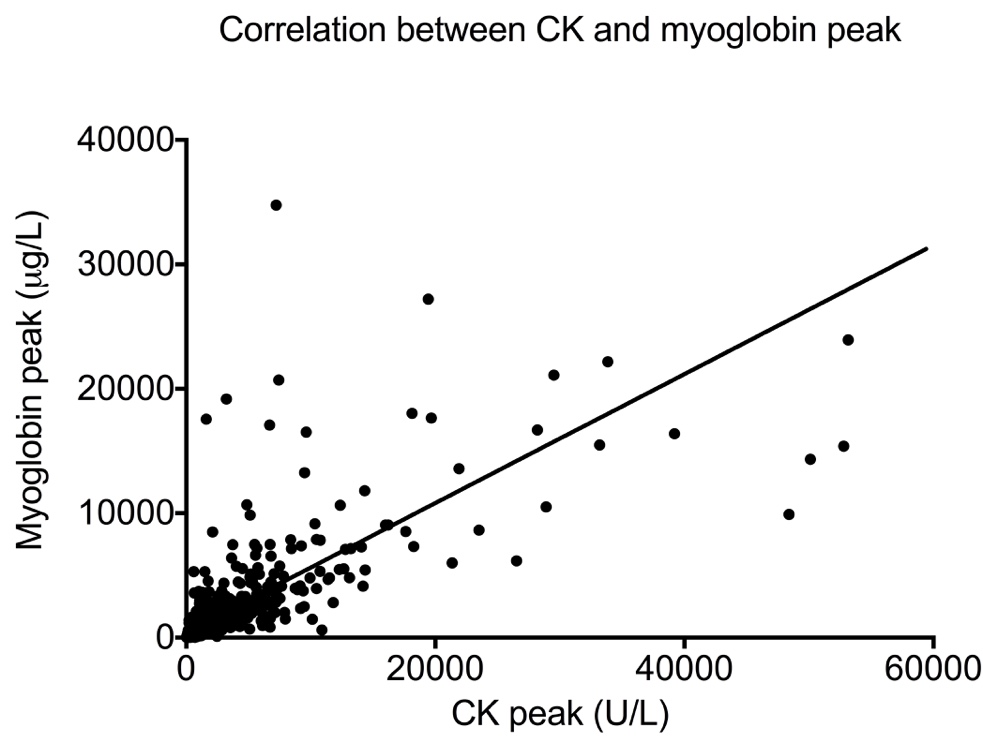


Slope=0.518, R^2^=0.34, p<0.001. 2 outliers are not reported in the graph because of CK level of 10000 U/L and myoglobin level of 121000 µg/L respectively.
