# Supplementary material for: Admission serum myoglobin and the development of acute kidney injury after major trauma
Source: Ann Intensive Care. 2021 Sep 24;11:140. doi: 10.1186/s13613-021-00924-3 (PMC8463647; doi:10.1186/s13613-021-00924-3)
Supplement: Supplementary file 7 — Additional file 7. Median and mean admission myoglobin levels according to ISS quartile. [file 13613_2021_924_MOESM7_ESM.docx]

**Additional file 7:** Median and mean admission myoglobin levels according to ISS quartile

| **ISS Quartile** | **Myoglobin, microg/L** |
| --- | --- |
| 1-9 (n=214) | 190 (82-356) |
| 9-17 (n=214) | 411 (206-469) |
| 17-29 (n=214) | 554 (203-1238) |
| 29-75 (n=215) | 1178 (483-3004) |

Median myoglobin according to ISS quartile

Mean myoglobin according to ISS quartile
