# Supplementary material for: Admission serum myoglobin and the development of acute kidney injury after major trauma
Source: Ann Intensive Care. 2021 Sep 24;11:140. doi: 10.1186/s13613-021-00924-3 (PMC8463647; doi:10.1186/s13613-021-00924-3)
Supplement: Supplementary file 8 — Additional file 8. Predictive performances of initial myoglobin and initial CK for acute kidney injury (KDIGO any stage) with baseline creatinine calculated with MDRD formula. [file 13613_2021_924_MOESM8_ESM.docx]

**Additional file 8:** Predictive performances of initial myoglobin and initial CK for acute kidney injury (KDIGO any stage) with baseline creatinine calculated with MDRD formula.

| **Parameter** | **Cut-off value** | **Sensitivity** | **Specificity** | **PPV** | **NPV** | **NLR** | **PLR** |
| --- | --- | --- | --- | --- | --- | --- | --- |
| **Prediction of AKI any KDIGO stage** | | | | | | | |
| Admission myoglobin | 1039 | 61 (51-69) | 76 (72-79) | 25 (20-31) | 93 (91-95) | 0.5 (0.4-0.7) | 2.5 (2.0-3.0) |
| Admission CK | 329 | 83 (75-89) | 40 (37-44) | 16 (13-19) | 95 (92-97) | 0.4 (0.3-0.6) | 1.4 (1.3-1.6) |
| **Prediction of AKI KDIGO 2 or 3** | | | | | | | |
| Admission myoglobin | 1217 | 74 (64-81) | 77 (74-80) | 29 (24-35) | 96 (94-97) | 0.3 (0.2-0.5) | 3.2 (2.7-3.8) |
| Admission CK | 842 | 81 (72-87) | 59 (56-63) | 21 (17-25) | 96 (94-97) | 0.3 (0.2-0.5) | 2.0 (1.7-2.3) |

CK = creatine kinase, KDIGO = kidney disease improving global outcomes, NLR = negative likelihood ratio, NPV = negative predictive value, PLR = positive likelihood ratio, PPV = positive predictive value.*p<0.05 (Chi square test).
