# Supplementary material for: Admission serum myoglobin and the development of acute kidney injury after major trauma
Source: Ann Intensive Care. 2021 Sep 24;11:140. doi: 10.1186/s13613-021-00924-3 (PMC8463647; doi:10.1186/s13613-021-00924-3)
Supplement: Supplementary file 9 — Additional file 9. Calibration of multivariable models as assessed by the Hosmer–Lemeshow statistical test. [file 13613_2021_924_MOESM9_ESM.docx]

**Additional file 9:** Calibration of multivariable models as assessed by the Hosmer-Lemeshow statistical test

|  | **Model 1** | | | **Model 2** | | |
| --- | --- | --- | --- | --- | --- | --- |
|  | **Base** | **Myoglobin** | **CK** | **Base** | **Myoglobin** | **CK** |
| **Baseline creatinine = creatinine calculated with MDRD** | | | | | | |
| **Hosmer-Lemeshow** | 0.112 | 0.112 | 0.265 | 0.152 | 0.457 | 0.151 |
| **Baseline creatinine = creatinine on admission** | | | | | | |
| **Hosmer-Lemeshow** | 0.926 | 0.677 | 0.969 | 0.05 | 0.110 | 0.090 |
| **Baseline creatinine = lowest creatinine over the first 5 days** | | | | | | |
| **Hosmer-Lemeshow** | 0.061 | 0.150 | 0.189 | 0.005 | 0.190 | 0.200 |

Results of the Hosmer-Lemeshow statistical test are given as p-values. Base model 1 is the model established by Haynes et al.[21] that includes age, admission phosphate, admission creatinine and hemorrhagic shock as independent variables. Base model 2 is the model established by Harrois et al. [4] that includes maximum prehospital heart rate, minimum systolic blood pressure, admission lactate, injury severity score (ISS) and hemorrhagic shock. Models were constructed for 3 AKI definitions that differ according to baseline creatinine definition. CK = creatine kinase, MDRD = Modification of Diet in Renal Disease
