# Supplementary material for: Admission serum myoglobin and the development of acute kidney injury after major trauma
Source: Ann Intensive Care. 2021 Sep 24;11:140. doi: 10.1186/s13613-021-00924-3 (PMC8463647; doi:10.1186/s13613-021-00924-3)
Supplement: Supplementary file 10 — Additional file 10. Performances of variable from multivariable models to predict acute kidney injury (KDIGO any stage). [file 13613_2021_924_MOESM10_ESM.docx]

| **Variable** | **AUC-ROC** | **Optimal cut-off** | **Sensitivity** | **Specificity** | **PPV** | **NPV** | **PLR** | **NLR** |
| --- | --- | --- | --- | --- | --- | --- | --- | --- |
| **Age** | 0.726 (0.672-0.779) | 54 | 55 (45-64) | 81 (78-84) | 28 (22-35) | 93 (91-95) | 2.92 (2.32-3.67) | 0.50 (0.45-0.69) |
| **Admission lactate** | 0.734 (0.690-0.798) | 2.6 | 61 (51-69) | 75 (72-79) | 30 (24-36) | 93 (90-95) | 2.59 (2.11-3.19) | 0.48 (0.37-0.63) |
| **Admission creatinine** | 0.633 (0.591-0.675) | 80 | 61 (55-67) | 62 (58-66) | 44 (39-49) | 77 (73-80) | 1.60 (1.39-2.78) | 0.63 (0.53-0.74) |
| **Admission phosphate** | 0.650 (0.608-0.691) | 1.12 | 48 (42-54) | 79 (76-82) | 52 (46-58) | 76 (72-79) | 2.28 (1.86-3.60) | 0.66 (0.59-0.75) |
| **Minimum prehospital MAP** | 0.714 (0.652-0.777) | 71 | 59 (49-68) | 79 (76-82) | 27 (21-33) | 93 (91-95) | 2.78 (2.24-3.45) | 0.52 (0.41-0.67) |
| **ISS** | 0.741 (0.690-0.795) | 25 | 71 (62-80) | 65 (62-68) | 25 (21-30) | 94 (92-96) | 2.51 (2.14-2.96) | 0.38 (0.27-0.52) |
| **Prehospital maximum heart rate** | 0.523 (0.449-0.596) | 117 | 30 (22-40) | 87 (84-89) | 24 (17-32) | 90 (88-92) | 2.32 (1.64-3.28) | 0.80 (0.70-0.91) |

**Additional file 10**: Performances of variables included in models 1 and 2 to predict AKI KDIGO any stage (stage 1, 2 or 3)

Performance parameters are given with their 95% confidence interval. AUC-ROC = Area under the receiver operating characteristic curve, ISS=injury severity score, MAP=mean arterial pressure, NLR=Negative likelihood ratio, NPV=Negative predictive value, PLR=positive likelihood ratio, PPV=positive predictive value.
