# Supplementary material for: Admission serum myoglobin and the development of acute kidney injury after major trauma
Source: Ann Intensive Care. 2021 Sep 24;11:140. doi: 10.1186/s13613-021-00924-3 (PMC8463647; doi:10.1186/s13613-021-00924-3)
Supplement: Supplementary file 11 — Additional file 11. Performances of variable from multivariable models to predict acute kidney injury (KDIGO stage 2 or 3). [file 13613_2021_924_MOESM11_ESM.docx]

**Additional file11**: Performances of variables included in models 1 and 2 to predict AKI KDIGO stage 2 or 3

| **Variable** | **AUC-ROC** | **Optimal cut-off** | **Sensitivity** | **Specificity** | **PPV** | **NPV** | **PLR** | **NLR** |
| --- | --- | --- | --- | --- | --- | --- | --- | --- |
| **Age** | 0.748 (0.664-0.832) | 54 | 61 (44-75) | 78 (75-81) | 10 (7-15) | 98 (97-99) | 2.81 (2.07-3.80) | 0.50 (0.33-0.77) |
| **Admission lactate** | 0.778 (0.701-0.856) | 2.6 | 74 (57-86) | 72 (69-75) | 11 (8-16) | 98 (97-99) | 2.66 (2.09-3.39) | 0.36 (0.20-0.65) |
| **Admission creatinine** | 0.714 (0.650-0.779) | 97 | 54 (44-63) | 85 (82-87) | 32 (25-39) | 93 (91-95) | 3.53 (2.75-4.53) | 0.54 (0.44-0.68) |
| **Admission phosphate** | 0.750 (0.693-0.804) | 1.12 | 67 (57-75) | 78 (74-80) | 28 (23-34) | 95 (93-96) | 2.97 (2.45-3.60) | 0.43 (0.32-0.57) |
| **Minimum prehospital MAP** | 0.745 (0.650-0.841) | 74 | 73 (56-86) | 71 (68-74) | 9 (6-13) | 99 (97-99) | 2.51 (1.97-3.19) | 0.38 (0.21-0.68) |
| **ISS** | 0.767 (0.685-0.850) | 20 | 88 (73-95) | 57 (53-60) | 7 (5-11) | 99(98-100) | 2.02 (1.74-2.35) | 0.21 (0.09-0.54) |
| **Prehospital maximum heart rate** | 0.533 (0.421-0.646) | 120 | 27 (15-44) | 87 (84-89) | 8 (4-14) | 97 (95-98) | 2.08 (1.16-3.73) | 0.84 (0.68-1.03) |

Performance parameters are given with their 95% confidence interval. AUC-ROC = Area under the receiver operating characteristic curve, ISS=injury severity score, MAP=mean arterial pressure, NLR=Negative likelihood ratio, NPV=Negative predictive value, PLR=positive likelihood ratio, PPV=positive predictive value.
