# Supplementary material for: Admission serum myoglobin and the development of acute kidney injury after major trauma
Source: Ann Intensive Care. 2021 Sep 24;11:140. doi: 10.1186/s13613-021-00924-3 (PMC8463647; doi:10.1186/s13613-021-00924-3)
Supplement: Supplementary file 12 — Additional file 12. Continuous net reclassification improvement (NRI) and IDI when adding admission myoglobin and CK to 2 established models of post-traumatic AKI prediction. [file 13613_2021_924_MOESM12_ESM.docx]

**Additional file 12:** Continuous net reclassification improvement (NRI) and IDI when adding admission myoglobin and CK to 2 established models of post-traumatic AKI prediction

|  | **Model 1** | | | **Model 2** | | |
| --- | --- | --- | --- | --- | --- | --- |
|  | **Base** | **Myoglobin** | **CK** | **Base** | **Myoglobin** | **CK** |
| **Baseline creatinine = creatinine calculated with MDRD** | | | | | | |
| **NRI (95% CI)** | - | 0.325 (0.121-0.528) | - | - | 0.298 (0.094-0.502) | - |
| **p-value NRI** | - | 0.002 | - | - | 0.004 | - |
| **NRI_event_** | - | -0.098 (-0.292-0.096) | - | - | -0.098 (-0.286-0.095) | - |
| **p-value NRI_event_** | - | 0.172 | - | - | 0.177 | - |
| **NRI_non-event_** | - | 0.423 (0.360-0.486) | - | - | 0.396 (0.329-0.360) | - |
| **p-value NRI_non-event_** | - | <0.001 | - | - | <0.001 | - |
| **IDI** | - | 0.008 (-0.003-0.018) | - | - | 0.027 (0.006-0.048) | - |
| **p-value IDI** | - | 0.143 | - | - | 0.01 | - |
| **Baseline creatinine = creatinine on admission** | | | | | | |
| **NRI (95% CI)** | - | 0.201 (0.001-0.403) | - | - | - | - |
| **p-value NRI** | - | 0.050 | - | - | - | - |
| **NRI_event_** | - | -0.176(-0.369-0.019) | - | - | - | - |
| **p-value NRI_event_** | - | 0.086 | - | - | - | - |
| **NRI_non-event_** | - | 0.377 (0.311-0.342) | - | - | - | - |
| **p-value NRI_non-event_** | - | <0.001 | - | - | - | - |
| **IDI** | - | 0.011 (-0.005-0.028) | - | - | - | - |
| **p-value IDI** | - | 0.185 | - | - | - | - |
| **Baseline creatinine = lowest creatinine over the first 5 days** | | | | | | |
| **NRI (95% CI)** | - | 0.323 (0.119-0.527) | 0.158 (-0.042-0.358) | - | 0.381 (0.176-0.586) | 0.097 (-0.185-0.205) |
| **p-value NRI** | - | 0.002 | 0.121 | - | <0.001 | 0.922 |
| **NRI_event_** | - | -0.078(-0.265-0.123) | -0.235(-0.420--0.237) | - | 0.020 (-0.169-0.208) | -0.333 (-0.515—0.144) |
| **p-value NRI_event_** | - | 0.229 | 0.02 | - | 0.620 | 0.002 |
| **NRI_non-event_** | - | 0.401(0.339-0.464) | 0.393(0.327-0.459) | - | 0.361(0.293-0.426) | 0.343 (0.279-0.408) |
| **p-value NRI_non-event_** | - | <0.001 | <0.001 | - | <0.001 |  |
| **IDI** | - | 0.036 (0.001-0.072) | -0.006 (-0.026-0.012) | - | 0.039 (0.015-0.064) | 0.002 (-0.011-0.014) |
| **p-value IDI** | - | 0.04 | 0.513 | - | 0.002 | 0.780 |

Base model 1 is the model established by Haynes et al.^4^ that includes age, admission phosphate, admission creatinine and hemorrhagic shock as independent variables. Base model 2 is the model established by Harrois et al. ^3^ that includes maximum prehospital heart rate, minimum systolic blood pressure, admission lactate, injury severity score (ISS) and hemorrhagic shock. Models were constructed for 3 AKI definitions that differ according to baseline creatinine definition. NRI and IDI were calculated only if the model coefficient of myoglobin or CK was statistically significant. CK = Creatine Kinase, CI = Confidence Interval, IDI = Integrated Discrimination Improvement, MDRD = Modification of Diet in Renal Disease.

NRI_event_ = proportion of AKI subjects with an increased risk of AKI (as calculated in the model including myoglobin versus the base model) - proportion of AKI subjects with a decreased risk of AKI (in the model including myoglobin versus the base model)

NRI_non-event_ = proportion of non-AKI subjects with a decreased risk of AKI (as calculated in the model including myoglobin (or CK) versus the base model) - proportion of non-AKI subjects with an increased risk of AKI (in the model including myoglobin (or CK) versus the base model)

NRI= continuous Net Reclassification Improvement. NRI is equal to (NRI_event_ + NRI_non-event_).
